# Undiagnosed Cryptic Diversity in Small, Microendemic Frogs (*Leptolalax*) from the Central Highlands of Vietnam

**DOI:** 10.1371/journal.pone.0128382

**Published:** 2015-05-28

**Authors:** Jodi J. L. Rowley, Dao T. A. Tran, Greta J. Frankham, Anthony H. Dekker, Duong T. T. Le, Truong Q. Nguyen, Vinh Q. Dau, Huy D. Hoang

**Affiliations:** 1 Australian Museum Research Institute, Australian Museum, Sydney, New South Wales, Australia; 2 Faculty of Biology, University of Science-Ho Chi Minh City, Ho Chi Minh City, Vietnam; 3 Federation University (Ballarat), Mt Helen, Victoria, Australia; 4 Institute of Ecology and Biological Resources, Vietnam Academy of Science and Technology, Cau Giay, Hanoi, Vietnam; University of Colorado, UNITED STATES

## Abstract

A major obstacle in prioritizing species or habitats for conservation is the degree of unrecognized diversity hidden within complexes of morphologically similar, “cryptic” species. Given that amphibians are one of the most threatened groups of organisms on the planet, our inability to diagnose their true diversity is likely to have significant conservation consequences. This is particularly true in areas undergoing rapid deforestation, such as Southeast Asia. The Southeast Asian genus *Leptolalax* is a group of small-bodied, morphologically conserved frogs that inhabit the forest-floor. We examined a particularly small-bodied and morphologically conserved subset, the *Leptolalax applebyi* group, using a combination of molecular, morphometric, and acoustic data to identify previously unknown diversity within. In order to predict the geographic distribution of the group, estimate the effects of habitat loss and assess the degree of habitat protection, we used our locality data to perform ecological niche modelling using MaxEnt. Molecular (mtDNA and nuDNA), acoustic and subtle morphometric differences revealed a significant underestimation of diversity in the *L*. *applebyi* group; at least two-thirds of the diversity may be unrecognised. Patterns of diversification and microendemism in the group appear driven by limited dispersal, likely due to their small body size, with several lineages restricted to watershed basins. The *L*. *applebyi* group is predicted to have historically occurred over a large area of the Central Highlands of Vietnam, a considerable portion of which has already been deforested. Less than a quarter of the remaining forest predicted to be suitable for the group falls within current protected areas. The predicted distribution of the *L*. *applebyi* group extends into unsurveyed watershed basins, each potentially containing unsampled diversity, some of which may have already been lost due to deforestation. Current estimates of amphibian diversity based on morphology alone are misleading, and accurate alpha taxonomy is essential to accurately prioritize conservation efforts.

## Introduction

The current biodiversity crisis urgently requires the delineation of conservation priorities for both species and habitats [1–3]. One of the first steps towards conservation prioritization is to measure and map biodiversity, but our understanding of species diversity, and how it varies spatially, is limited [4,5]. A major obstacle in understanding biodiversity is the extent of unrecognized diversity hidden within complexes of morphologically similar species [6]. Speciation is not always accompanied by morphological change, and our inability to recognize these “cryptic” species may mask true species diversity, with serious conservation consequences [6–9].

Given that amphibians are one of the most threatened groups of organisms on the planet [10], our inability to diagnose and map their true diversity is likely to have significant consequences for their conservation [7]. Since cryptic species are likely to have smaller geographical ranges than the previously recognized ‘species’, and small range size generally is the strongest predictor of a species’ risk of extinction, they are likely to be more threatened [11–13]. If they remain undetected, there is a risk that they will become extinct without our knowledge [14].

It is becoming increasingly apparent that a significant proportion of amphibian diversity remains hidden within unrecognized, cryptic species complexes. Amphibians commonly exhibit conservative morphological evolution that may conceal true species diversity [9, 15–19]. This is likely due in part to their dependence non-visual (acoustic) cues for species recognition and mate choice. As such, molecular and bioacoustic tools are often required to reveal amphibian cryptic species (e.g. [13, 16, 20–22]).

A significant proportion the amphibian species diversity in Southeast Asia appears to be hidden within cryptic species groups. To date, every molecular study examining widespread Southeast Asian amphibian species throughout their ranges has revealed unrecognized species diversity [13, 23–25]. The majority of this diversity is found only in intact forest [13], an alarming fact given that the region has the highest relative deforestation rate of any major tropical region [26, 27]. The apparently high level of undiagnosed diversity, coupled with ongoing deforestation, raises the possibility that Southeast Asia will lose species of amphibian before they are discovered.

The genus *Leptolalax* Dubois 1983 is a group of small, morphologically conserved frogs that inhabit the forest floor and rocky streams in hilly evergreen forest throughout Southeast Asia, southern China and northeastern India [28]. Most of the 41 known *Leptolalax* species are small (<60 mm SVL) and brown, resembling their forest floor substrate, and lack striking interspecific differences in their appearance. Their habitat is often difficult to access and their advertisement calls faint and insect-like (e.g. [29–31]). As a result, *Leptolalax* are both difficult to detect in the field and to identify to species. Increased use of acoustic and molecular data in delineating species boundaries in the group, along with additional survey effort, has resulted in more than doubling of the known species since 2000 [28].

**Fig 1:**
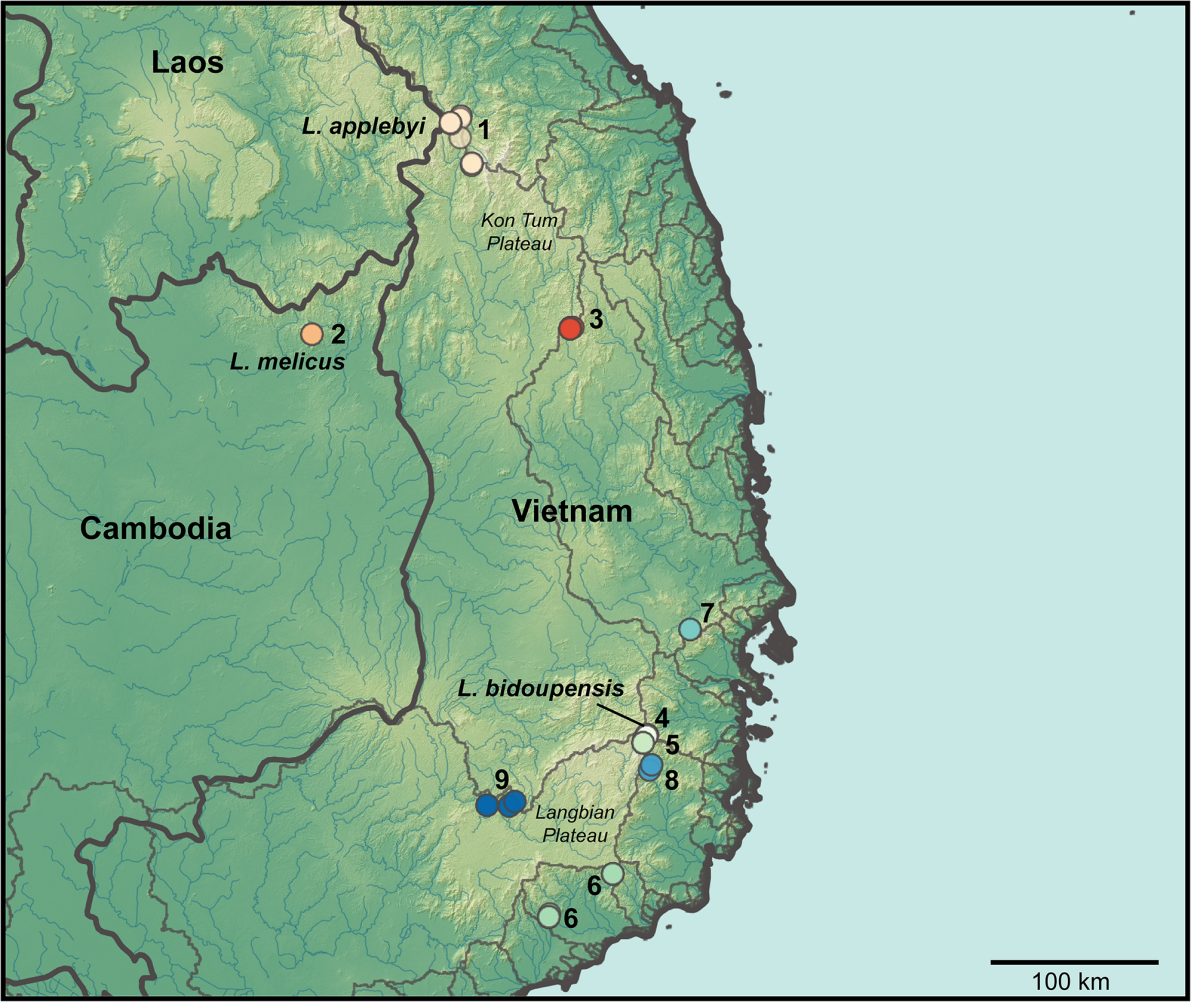
Map showing the localities where specimens in the *Leptolalax applebyi* group were collected. Colours of localities assigned based on the nine molecular lineages. Paler areas are higher elevation. Dark grey lines show country boundaries, pale grey lines show watershed boundaries and blue lines show rivers.

Three species of very small *Leptolalax* (<30 mm SVL) have been described in recent years from the Central Highlands (or the Tay Nguyen Plateau) of Vietnam and adjacent northeastern Cambodia: *Leptolalax applebyi* Rowley & Cao 2009 from the Kon Tum Plateau in the north of the Highlands, *L*. *bidoupensis* Rowley *et al*. 2011 from the south of the Highlands, in the Langbian Plateau, and *L*. *melicus* Rowley *et al*. 2010 from northwestern slopes of the Highlands in northeastern Cambodia (Fig 1). These three species, the ‘*L*. *applebyi* group’, are distinguished from all other species of *Leptolalax* by a combination of their very small size, quiet, rasping advertisement calls, morphological similarity (including a dark brownish red ventral surface with white speckling and black markings on the flanks) and mitochondrial DNA [29–32]. They also share similar breeding habitat, preferring the trickles at headwaters of small mountain streams and seeps, as opposed to the larger streams that the other *Leptolalax* are associated with (J. Rowley, pers. obs.). Given their likely limited dispersal potential due to their small size and highly localized breeding habitat, low detectability, and complex topography within their range, it is likely the *L*. *applebyi* group contains undiagnosed species diversity.

This study surveyed additional areas of the Central Highlands of Vietnam and northeastern Cambodia and identified a number of new populations of frogs assignable to the *L*. *applebyi* group. Specimens, tissue samples and advertisement calls were sampled from all new populations to aid in identifying undiagnosed diversity. Advertisement calls have been particularly important in delineating species boundaries in the genus (e.g. [29–31]), and more broadly have been shown to serve as premating species isolating mechanisms, even when the morphologies of the frogs are indistinguishable (e.g. [33]). Additionally, we used these new localities in ecological niche modelling (e.g. [34]) in order to predict the geographic distribution of the group, and estimate historical habitat loss and current degree of habitat protection for the *L*. *applebyi* group.

## Materials and Methods

### Ethics Statement

All voucher specimens were obtained under appropriate permits for each site. Protocols for collection of specimens by JR were approved by the approved by the Australian Museum Animal Ethics Committee (#09–01); voucher specimens were euthanized using benzocaine. None of the species collected for this study are listed as protected, threatened, or listed under the Convention on International Trade in Endangered Species of Wild Fauna and Flora—CITES (www.cites.org).

The National Forestry Protection Department, Diep Thanh Phong and all at Quang Nam Forestry Protection Department gave permission for surveys in Quang Nam Province, Vietnam. Staff of Ngoc Linh Nature Reserve issued permissions (Permit numbers 776/BNN-KL, 748/BNNKL) and the People’s Committee of Kon Tum Province issued permit number 548/UBND-DN for work in Kon Tum Province, Vietnam. H.E. Dr Mok Mareth, Senior Minister of Environment and H.E. Chay Samith, General Director of Administration for Nature Conservation and Protection permitted us to conduct surveys and issued specimen export permits (Permit # 405 DNCP MoE [transport]) in Modulkiri Province, Cambodia. The Vietnamese Ministry of Agriculture and Rural Development issued permission to collect (Permit number 3023/GT-BNN-KL & 1430/GT-BNN-KL) for Lam Dong Province, Vietnam. The Vietnamese Ministry of Agriculture and Rural Development (Permit number 779/TCLN-BTTN and Export Permit No. 11VN0003N/KL-NC) for work in Binh Thuan Province. Mr Nguyen Cong Van, Director of Phuoc Binh National Park permitted us to work in Ninh Thuan Province, Vietnam (University of Science Ho Chi Minh City document 425/KHTN-KH and 494/KHTN-KH).

### Surveys

*Leptolalax* specimens were collected between 2006 and 2011 from 22 sites (defined as localities >1 km apart) in eight provinces within in the Central Highlands of Vietnam and a single province in adjoining northeastern Cambodia. These localities fell within two distinct regions; central Vietnam and northeastern Cambodia (the Kon Tum Plateau), and southern Vietnam (the Langbian Plateau) (Fig 1). Sites fell within seven watershed basins and were all within broadleaf evergreen forest between 200–1935 m asl. Most frogs were encountered during nocturnal surveys at breeding sites, where the advertisement calls of males increased detectability. Geographic coordinates were obtained using a Garmin GPSMAP 60CSx GPS receiver and recorded in datum WGS 84.

A total of 87 adult male and 19 adult female specimens were collected. Prior to preservation, tissues (liver or thigh muscle) was removed for molecular analysis and stored in EDTA/DMSO or 80% ethanol. Specimens were then fixed in 10% formalin and then stored in 70% ethanol or fixed and stored in 80% ethanol. Specimens were deposited at the American Museum of Natural History (AMNH), Australian Museum (AMS), Institute of Ecology and Biological Resources (IEBR), Museum of Vertebrate Zoology, University of California Berkeley (MVZ), North Carolina Museum of Natural Sciences (NCSM), University of Science, Ho Chi Minh City (UNS), and Zoologisches Forschungsmuseum Alexander Koenig (ZMFK), (S1 Table). Type specimens of *Leptolalax applebyi*, *L*. *bidoupensis* and *L*. *melicus* were included in all morphometric, molecular and acoustic analyses.

### Molecular data

Genomic DNA was extracted from EDTA/DMSO or ethanol preserved tissues using DNeasy Blood and tissue kit (QIAGEN GmbH, Hilden, Germany), using the protocols for purification of total DNA from animal tissues. Two mitochondrial (mtDNA) and three nuclear DNA (nuDNA) regions were amplified for this study. The two mtDNA regions were 16S, using the primers (5’-3’) 16AR CGCCTGTTTATCAAAAACAT and 16AB CCGGTCTGAACTCAGATCACGT [35] and Cytochrome *b*, using the primers L14841 AAAAAGCTTCCATCCAACATCTCAGCATGATGAAA and H15149 AAACTGCAGCCCCTCAGAATGATATTTGTCCTCA [36]. The three nuDNA regions were; NTF3, using the primers NTF3F3 TCTTCCTTATCTTTGTGGCATCCACGCTA and NTF3_R ACATTGRGAATTCCAGTGTTTGTCGTCA [37], NCX, using the primers NCX_1F ACAACAGTRAGRATATGGAA and NCX_1R CCTTCTGTTTCRATGATCAT [38] and SLC8A3 using the primers SCF_1F CCATAGARGTCATAACATCACA and SCF_1R TTCATRACYTTGCCRTCCAT [38].

PCR amplification was carried out in 25μl reactions with 1000 ng of genomic DNA, 1 x Reaction Buffer (Bioline My Taq Red Reagent Buffer), 2 pmol corresponding primers and Bioline My Taq Red DNA polymerase (0.5 unit). Negative controls were included in each PCR. Thermocycling was performed on an Eppendorf Mastercycler EpS (Eppendorf, Hamburg, Germany) under the following conditions: Cytochrome *b* and 16S initial denaturation 94°C (2 mins), 2 cycles of 94°C (20s) denaturation, 52°C (40s) annealing and 72°C(60s) extension, followed by 33 cycles of 94°C (20s) denaturation, 50°C (40s) annealing and 72°C (50s) extension, followed by a final extension step at 72°C for 5 mins. For NTF3 the following conditions were used; 94°C (5 mins), 35 cycles of 94°C (45s) denaturation, 60°C (30s) annealing and 72°C (60s) extension, with a final extension step at 72°C for 7 mins. For NCX and SLC8A3 the conditions were; 94°C (3 mins), 35 cycles of 94°C (60s) denaturation, 50°C (60s) annealing and 72°C (60s) extension, and a final extension step at 72°C for 3 mins. All PCR products were purified using Exo-Sap-IT (USB Corporation, OH, USA), and directly sequenced at the Australian Genome Research Facility (Sydney, Australia) and Macrogen (Seoul, Korea).

Sequences were edited with reference to chromatograms using Sequencher v. 4.10 (Gene Codes, Ann Arbor, MI, USA) and deposited in GenBank (accession # KR018001–KR018126; S1 Table). The data set was aligned using the ClustalW option in MEGA 5 [39].

Phylogenetic relationships among individuals were estimated using Bayesian Inference conducted in Mr Bayes version 3.1 [40]. An appropriate model of evolution was determined for each gene region using jModelTest version 1.1 [41, 42] and the Akaike Information Criterion (AIC). Analyses were conducted using default settings for priors, one for the concatenated nuDNA (unphased), partitioned by gene region, and the other for the concatenated mtDNA, partitioned by gene region. MCMC sampling was used to calculate posterior probability. Two independent analyses ran simultaneously with four chains per run (1 cold, 3 hot). The chains were run for 10,000,000 generations and sampled every 100 generations, generating 100,000 sampled trees. Individual gene trees were also generated using the above methods, with 100 000 generations run sampled every 100 generations. Tracer version 1.5 [43] was used to determine that chain convergence and adequate Effective Sample Size (>100) were obtained. Posterior probabilities (decimals) were used to assess the level of branch support. Values ≥ 0.90 were considered significant. *Leptolalax* species that are not considered part of the *L*. *applebyi* group (*Leptolalax aereus*, *L*. *bourreti*, and *L*. *firthi* [32]), along with closely related megophrid genera were used as outgroups (*Brachytarsophrys intermedia*, *Leptobrachium chapaense*, and *Oreolalax sterlingae*).

To resolve individual alleles at the nuclear loci NTF3, NCX and SLC8A3 for heterozygous individuals and investigate allele sharing, SeqPHASE [44] and PHASE [45] were used.

### Morphometric data

Morphometric data were taken from preserved specimens (to the nearest 0.1 mm) with digital callipers. We measured snout-vent length (SVL); head length from tip of snout to rear of jaws (HDL); head width at the commissure of the jaws (HDW); snout length from tip of snout to the anterior corner of eye (SNT); diameter of the exposed portion of the eyeball (EYE); interorbital distance (IOD); horizontal diameter of tympanum (TMP); distance from anterior edge of tympanum to posterior corner of the eye (TEY); and tibia length with the hindlimb flexed (TIB), manus length from tip of third digit to base of inner palmar tubercle (ML), pes length from tip of fourth toe to base of the inner metatarsal tubercle (PL), maximum diameter of pectoral gland (PEC), maximum diameter of femoral gland (FEM), and maximum diameter of humeral gland (HUM). Sex was determined by direct observation of calling in life, the presence of internal vocal sac openings and/or gonadal inspection. See S2 Table for Museum registration numbers of specimens examined.

We performed Principal Component Analyses (PCA) based on original morphometric values for males, using varimax rotation. Due to the poor preservation of a number of specimens, we removed ML and PL from the analysis. To determine morphometric differences among molecular lineages, we examined each variable that contributed highly to the factor loadings (>0.80), removing the effect of SVL from all other variables. For variables with a normal distribution, we performed ANCOVA analyses, with SVL used as covariable, followed by Tukey HSD Post- Hoc tests for pairwise comparisons among the nine lineages identified molecularly. For non-normal variables, we removed the effect of SVL calculating ratios of the variable relative to SVL and performing nonparametric Wilcoxon/Kruskal-Wallis tests and Nonparametric Comparisons for each pair using the Wilcoxon Method. Statistical analyses were performed by using JMP software (ver. 10.0.2; SAS Institute Inc. Cary, NC, USA).

### Acoustic data

The use of bioacoustic data is useful in species delineation in anurans, as sexual selection may lead to the rapid divergence of advertisement calls, thereby generating prezygotic reproductive barriers [46–47]. We recorded the advertisement calls of individuals in the *L*. *applebyi* group with an Edirol R-09 WAVE/MP3 Recorder or a Zoom H4n Handy Mobile 4-Track recorder (44.1 kHz sampling rate, 24-bit encoding) and Røde NTG-2 condenser shotgun microphone. Calls were recorded at a distance of approximately 0.1–0.3 m and ambient temperatures were taken immediately after recordings using a Kestrel 3500 or 4000 hand-held weather meter.

Calls were analysed with Raven Pro 1.3 software (http://www.birds.cornell.edu/raven). Audiospectrograms in figures were calculated with fast-Fourier transform (FFT) of 1024 points, 50% overlap and 172 Hz grid-spacing, using Hanning windows. Comparisons of anuran advertisement calls in general are complicated by inconsistent use of terms and a lack of clear definitions in terminology. Here we use the definitions of Duellman [48], except that we define a single call as vocalisations produced during a single expiration [49]. Temporal and spectral parameters of calls were measured using the definitions of Cocroft & Ryan [50], except for fundamental frequency, where the definition of Duellman [48] was used. For each call recording, we measured the call duration (ms), intercall interval (ms), number of notes per call, internote interval (ms), percent of call composed of note 1, number of pulses per note and dominant frequency (kHz). We consider that structural differences (pulsed versus non pulsed, non- overlap in notes/call, number of pulses) in the advertisement calls indicative of prezygotic reproductive barriers (e.g. [51]).

### Ecological Niche Modelling

We modelled the potential distribution of the *Leptolalax applebyi* group using the R software toolkit, version 2.15.3 (R Core Team, 2013) and the “raster” (version 2.2–31) and “dismo” (version 0.9–3) packages. The latter was interfaced to version 3.3.3k of the MaxEnt program, a machine-learning algorithm that generates a cumulative probability distribution based on the principle of maximum entropy [52, 53]. The resulting output is an indicator of environmental suitability for the species, ranging from 0 (unsuitable) to 1 (optimal). DOMAIN [54], BIOCLIM [55], and generalized linear modelling methods were also used for comparison but resulted in lower AUC scores, consistent with previous studies [56, 57]

We used current climatic data from the WorldClim database (30-second grid; [58]). We defined our study area as a region 6° on each side containing our survey sites, from 104–110°E and 10–16°N. To account for correlations among the data, we performed a principal components analysis on the 19 WorldClim “bioclimatic variables” for all 518,400 grid cells in the study area. The first eight principal components were used, which together accounted for 99.98% of the variance in the data. Due to the association of species with streams in sloping terrain, we supplemented these eight climatic principal components with the slope of terrain, calculated from the WorldClim elevation data using the “terrain” function in R. The 1^st^, 3^rd^, and 6^th^ bioclimatic principal components, together with the slope, were most influential in the MaxEnt model. These three bioclimatic principal components loaded primarily on annual and wettest-quarter precipitation; on warmest-quarter vs wettest-quarter precipitation; and on a combination of eight temperature-related variables.

Occurrence records clustered into a northern (lineages 1–3) and southern (lineages 4–9) group. These clusters correspond to biogeographically distinct plateaus (e.g. average annual rainfall 2151–3032 mm at the northern sites compared to 1292–2119 mm at southern sites), separated by low-elevation terrain. To account for the heterogeneity in climatic niches, we carried out ecological niche modelling separately for each geographic clusters identified, and then combined results (following a similar method to [59]). The predicted range for the *Leptolalax applebyi* group was the union of the two predicted cluster ranges. The 106 specimens from the *Leptolalax applebyi* group were found at 11 distinct northern sites, and 20 distinct southern sites, so that ecological niche modeling was performed using 11 and 20 “presence” datapoints respectively. MaxEnt modeling performs acceptably well with as few as 5–10 datapoints [60, 61].

As usual [62], the species “presence” data in each case was supplemented with 1,000 randomly chosen “background” datapoints. We chose these to be at least 5 km from “presence” sites. As for the “presence” data, the values of the terrain slope and the eight climatic principal components at these datapoints was used. Model evaluation was conducted using a “leave out one third” process, in which two thirds of the “presence” data (randomly chosen) was used to train the model, and one third (together with 1,000 new “background” points) was used to test the model. This was performed 50 times, and median results chosen. The fit of models to the test data was evaluated using the test data sets by calculating the median area under the Receiver Operating Characteristic curve, or AUC [63]. On average, random guessing gives an AUC of 0.5, and a perfect model has an AUC of 1.0. Models with good predictive ability are generally accepted as having AUC scores >0.75 [64], and those with high accuracy scores of >0.9 [65]. The median value of the maximum of the sum of the sensitivity (true positive rate) and specificity (true negative rate) was used as a threshold for the suitability values calculated by MaxEnt. This thresholding technique generally works well, giving a balance between false positives and false negatives [66]. The two resulting distribution regions were then merged to give a predicted suitable range for the *L*. *applebyi* group.

In order to consider the effect of habitat loss on the geographic range of the species group, we obtained areas of Broadleaf Evergreen Forest, the forest type observed at all localities, from the GLCNMO Global Land Cover Map (derived from 2008 MODIS satellite imagery; [67]). Protected area boundaries were obtained from the World Database on Protected Areas (WPDA; [68]). Watershed boundaries were obtained from HydroSHEDS digital river database (30-second resolution; [69]).

Our sample size for each molecular lineage was too small to assess bioclimatic-niche space overlap in the *Leptolalax applebyi* group, (eg. [70, 71]), but possible separation of the identified molecular lineages in bioclimatic space was examined by plotting each of the 31 sites according to elevation (m), temperature seasonality, annual precipitation and precipitation seasonality (the latter three as extracted for each site from WorldClim “bioclimatic variables” [58]), factors known to strongly influence bioclimatic-niche divergence among species [70–72].

## Results

We collected a total of 106 specimens in the *Leptolalax applebyi* group, 87 adult males and 19 adult females, performed molecular analysis on 23 individuals from all main geographic areas, and analyzed the advertisement calls of 25 individuals from all but one main geographic area.

### Molecular

**Fig 2:**
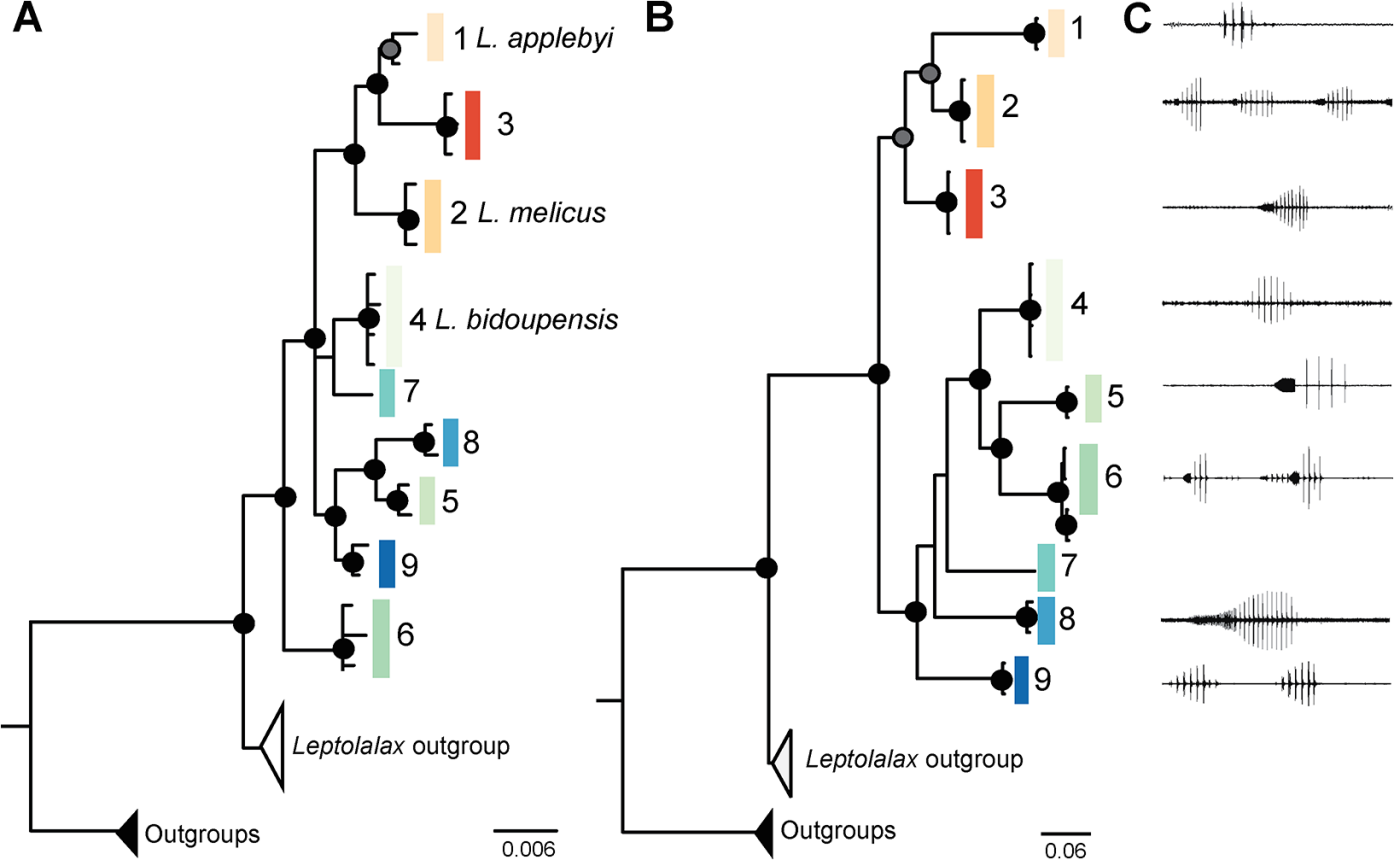
Bayesian inference (BI) trees based on (A) 1542 bp of nuclear gene sequences and (B) ~868 bp of mitochondrial gene sequences for *Leptolalax applebyi* group and outgroups. Node support is indicated by circles; those with Bayesian posterior probabilities of 0.95–0.99 are grey and 1.00 are black. (C) Two second waveform of relative amplitude over time for representative advertisement calls for mitochondrial lineages 1–6 and 8–9.

~868 bp of mtDNA and 1542 bp of nuDNA were obtained from 29 individuals (23 *L*. *applebyi* group, 3 *Leptolalax* species outside the *L*. *applebyi* group and 3 outgroups). When analyzed separately slightly different tree topologies were resolved from each gene region, however analysis of the concatenated mtDNA and nuDNA data resolved similar tree topologies, with nine distinct lineages identified in both data sets (Fig 2). No haplotypes were shared between individuals of different mtDNA lineages, and when the nuDNA was phased there were only two instances of individuals of different lineages sharing nuclear alleles (lineages 4 and 7 and lineages 9 and 5 in the NCX gene). Three of the molecular lineages identified corresponded with described species; lineage 1 (*L*. *applebyi*), lineage 2 (*L*. *melicus*), and lineage 4 (*L*. *bidoupensis*). Overall, the concatenated data set grouped the nine lineages into two larger lineages corresponding to geographic regions (a northern group centered on the Kon Tum Plateau and a southern group on the Langbian Plateau). The northern geographic lineages comprised lineages 1–3 (including *L*. *applebyi*, *L*. *melicus* and specimens from Gia Lai Province), whereas the southern cluster comprised lineages 4–9 (*L*. *bidoupensis* and remaining specimens from the Langbian Plateau).

The genetic differentiation within the group appears related to watershed basins, with lineages restricted to specific watershed basins or portions of specific watershed basins. While lineages 1, 4, and 9 appear to straddle watershed boundaries, lineages 2, 6 and 8 appear to be restricted to specific basins, and lineages 3 and 7 appear to be restricted to the northern and southern portions of a larger basin (Fig 1).

The percent uncorrected pairwise divergence at the 16S rRNA mitochondrial gene fragment examined ranged from 4.48–11.21% among lineages and 0.00–0.56% within lineages. Although we did not sample extensively within each watershed basin (i.e. many lineages were sampled from only a few nearby sites), there was evidence that divergence was related to barriers such as mountain ranges, rather than simply geographic distance. Specimens within lineage 6 came from over 43 km apart in the same drainage basin yet displayed only minor divergence at the 16S rRNA gene fragment (0.56%), while *L*. *bidoupensis* and lineage 5 displayed relatively high divergence (4.68%) despite being geographically separated by only 5 km.

### Morphometrics

**Fig 3:**
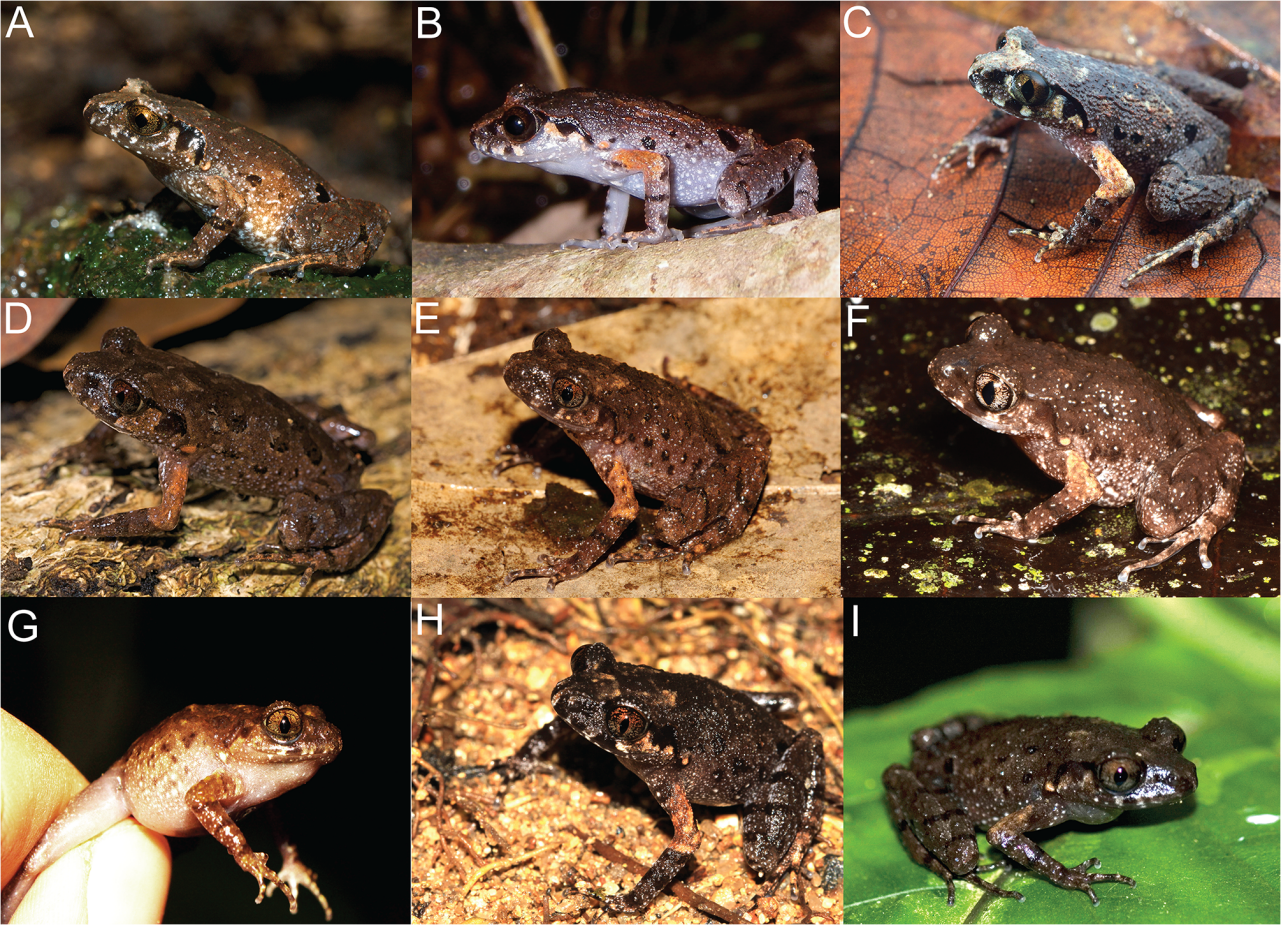
Images in life of frogs in the *Leptolalax applebyi* group. (A) *Leptolalax applebyi* (= molecular lineage 1; Kon Tum Province, Vietnam), (B) *Leptolalax melicus* (= molecular lineage 2; Ratanikiri Province, Cambodia), (C) *Leptolalax* sp. (= molecular lineage 3; Gia Lai Province, Vietnam), (D) *L*. *bidoupensis* (= molecular lineage 4; Lam Dong Province, Vietnam), (E) *Leptolalax* sp. (= molecular lineage 5; Lam Dong Province, Vietnam), (F) *Leptolalax* sp. (= molecular lineage 6; Binh Thuan Province; photo: Pedro Peloso), (G) *Leptolalax* sp. (= molecular lineage 7; Dak Lak Province, Vietnam), (H) *Leptolalax* sp. (= molecular lineage 8; Ninh Thuan Province, Vietnam), (I) *Leptolalax* sp. (= molecular lineage 9; Dak Nong Province, Vietnam).

**Fig 4:**
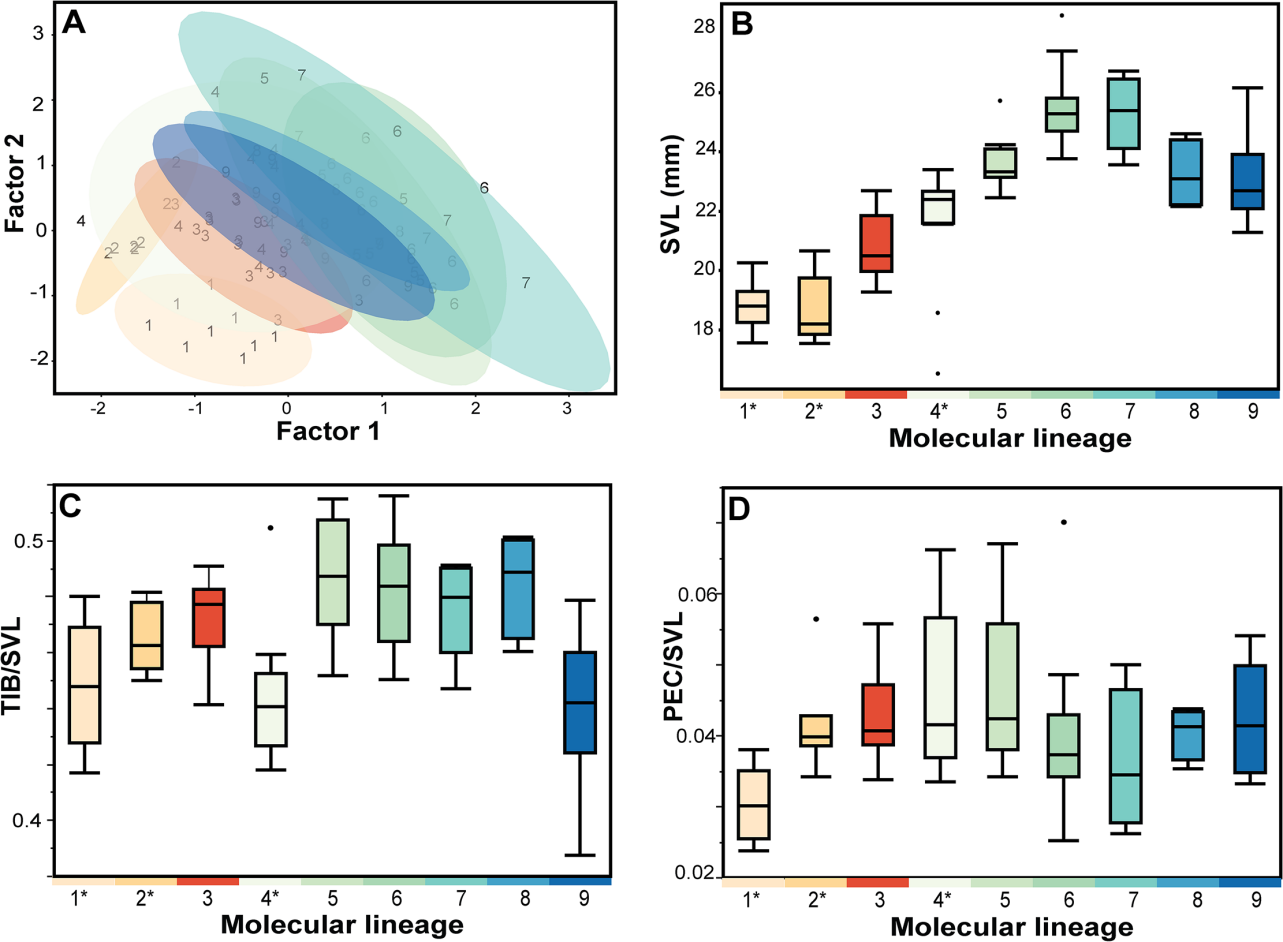
Morphometric differentiation among adult male frogs in each of the nine molecular lineages identified in *Leptolalax applebyi* group. (A) Scatterplots of the first two factors of Principal Component Analysis of male morphometrics. Points are numbered and 95% confidence ellipsoid coloured according to molecular lineages. Boxplots of (B) SVL, (C) relative tibia length (TIB/SVL) and (D) relative pectoral gland size (PEC/SVL) of adult males in each molecular lineage. For all boxplots, horizontal lines within each box represent the median and boxes encompass the 75th and 25th quartiles. Currently recognized species are marked with an asterix (1 = *L*. *applebyi*, 2 = *L*. *melicus*, 4 = *L*. *bidoupensis*).

Frogs were morphologically similar, all with a brown dorsum, dark lateral blotches and a dark supratympanic fold (Fig 3). Adult males ranged from 18.5–30.6 mm SVL and females from 21.7–32.1 mm. Principal component analyses (PCA) of our measurements for males reveal that the majority of the variance (60.1%) was explained by the first factor. This factor had high loadings for SVL, HDW, SNT, IOD, TMP, and TIB, suggesting that it is strongly influenced by body size (S4 Table). Factor 2, which is mostly influenced by PEC, explains 9.6% of the variance. Scatterplots of Factor 1 vs. Factor 2 shows separation between some of the lineages, especially *L*. *applebyi* (lineage 1) and other species (Fig 4A).

In order to translate these data into more diagnostically relevant information, we performed univariate analyses of those variables that were identified as most relevant by PCA (SVL, HDW, IOD, TMP, TIB and PEC). Only SVL (ANOVA; F = 36.0, p < 0.001), HDW (ANCOVA, F = 69.6, p = 0.0026), TIB/SVL (non-normal, Wilcox, Chi^2^ = 37.2, p < 0.001) and PEC/SVL (non-normal, Wilcox, Chi^2^ = 21.9, p < 0.0051) varied significantly among lineages. The most obvious difference between lineages was body size (Fig 4B), and almost all lineages can be differentiated morphologically from each other by a combination of SVL, HDW, TIB/SVL and PEC/SVL (Fig 4; Table 1). The two lineages geographically closest to each other (~5km), *L*. *bidoupensis* and Lineage 5, were clearly distinguished morphometrically, and all but Lineage 8 was morphometrically distinguishable from members of its geographically closest lineage (Table 1).

**Table 1:** Summary of morphological comparisons among males from each molecularly identified lineage of the *Leptolalax applebyi* group.

	Lineage 1 (*L*. *applebyi*)	Lineage 2 (*L*. *melicus*)	Lineage 3	Lineage 4 (*L*. *bidoupensis*)	Lineage 5	Lineage 6	Lineage 7	Lineage 8	Lineage 9
**Lineage 1 (*L*. *applebyi*)**									
**Lineage 2 (*L*. *melicus*)**	PEC								
**Lineage 3**	SVL, TIB, PEC	SVL							
**Lineage 4 (*L*. *bidoupensis*)**	SVL, HDW, PEC	SVL, TIB	TIB						
**Lineage 5**	SVL, HDW, TIB, PEC	SVL, TIB	SVL	SVL, TIB					
**Lineage 6**	SVL, TIB, PEC	SVL, TIB	SVL	SVL, TIB	SVL				
**Lineage 7**	SVL	SVL	SVL	SVL, TIB					
**Lineage 8**	SVL, HDW, PEC	SVL	SVL	TIB					
**Lineage 9**	SVL, PEC	SVL, TIB	SVL, TIB		TIB	SVL, TIB	SVL, TIB	TIB	

Morphometric variables that differed significantly between species. SVL was compared without covariate. To remove the effects of SVL on other variables, we used ANCOVAs with SVL used as covariable and Tukey’s HSD Post-Hoc analyses. For non-normal variables, we analysed ratios relative to SVL (TIB/SVL and PEC/SVL) using Wilcoxon/Kruskal-Wallis tests and Nonparametric Comparisons using the Wilcoxon Method. See methods for abbreviations.

### Acoustics

We obtained call recordings from 25 individuals representing all molecular lineages with the exception of lineage 7 (S3 Table). Calls were recorded at 12.9–26.4°C. Analysis was complicated by the relatively low number of individuals recorded, uneven sample size per lineage and the correlation of spectral and temporal call parameters to body size and temperature. Mean dominant frequency (kHz) was correlated with SVL (mm) (R^2^ = 62.1%, F = 27.91 p < 0.001), and mean internote interval (ms), mean note repetition rate (notes/s) and mean relative duration of note 1 (%) were correlated with temperature (R^2^ = 39.3%, F = 14.89 p = 0.0008; R^2^ = 34.2%, F = 11.93, p = 0.0022; R^2^ = 32.7%, F = 11.17, p = 0.0028).

**Fig 5:**
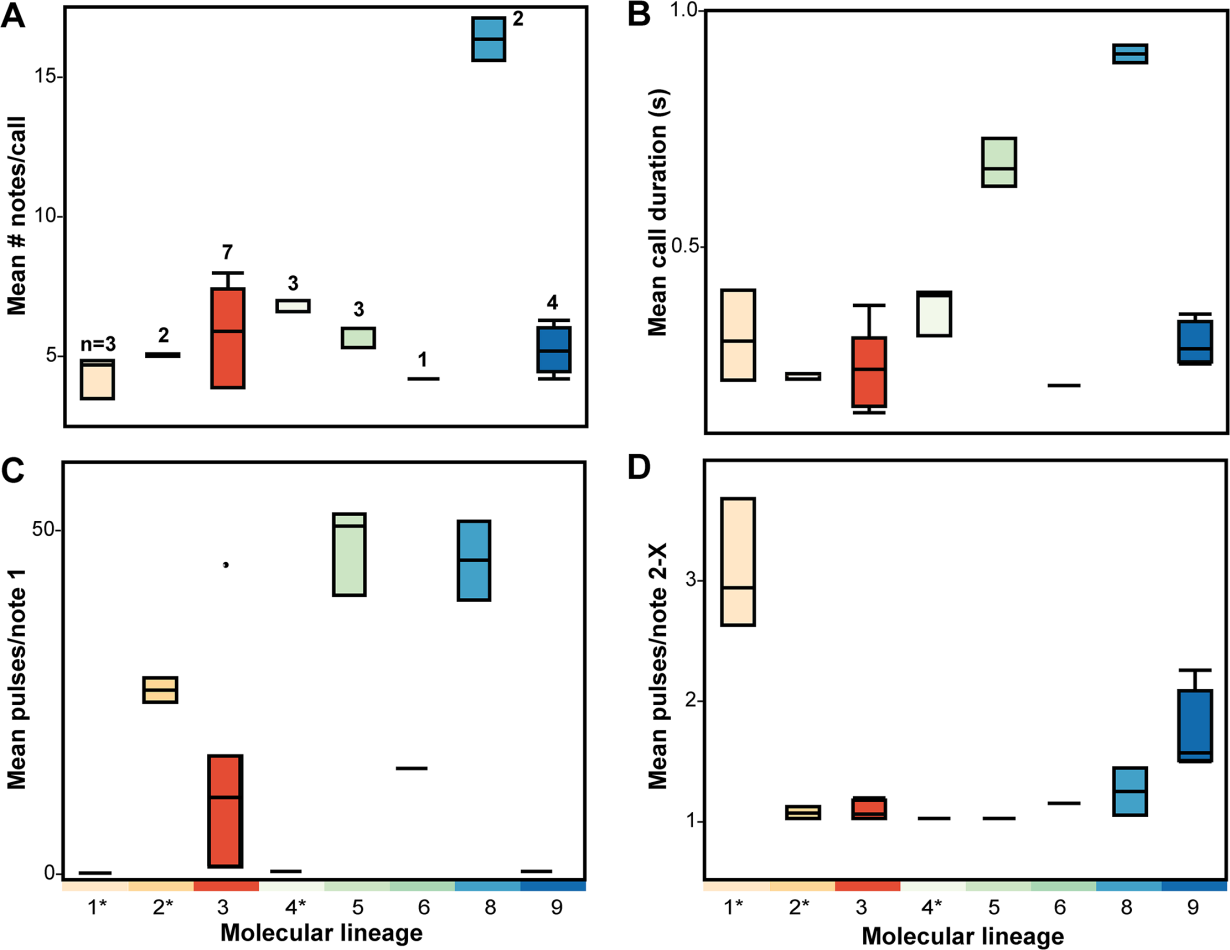
Boxplots of (A) mean number of notes per advertisement call, (B) mean call duration, (C) mean pulses in note 1 and (D) mean pulses in notes 2–X in frogs in each of the nine molecular lineages identified in *Leptolalax applebyi* group. Currently recognized species are marked with an asterix (1 = *L*. *applebyi*, 2 = *L*. *melicus*, 4 = *L*. *bidoupensis*). Vertical lines within each box represent the median and boxes encompass the 75th and 25th quartiles. Sample size varied each molecular lineage and is displayed on panel A. Measured variables did not vary significantly with SVL (mm) or temperature (°C).

Call structure varied considerably, with the advertisement calls of the lineages differing in terms of number of notes, the presence, duration and number of pulses in an introductory note, and pulse number in non-introductory notes (Figs 2 and 5).

### Ecological Niche Modelling

**Fig 6:**
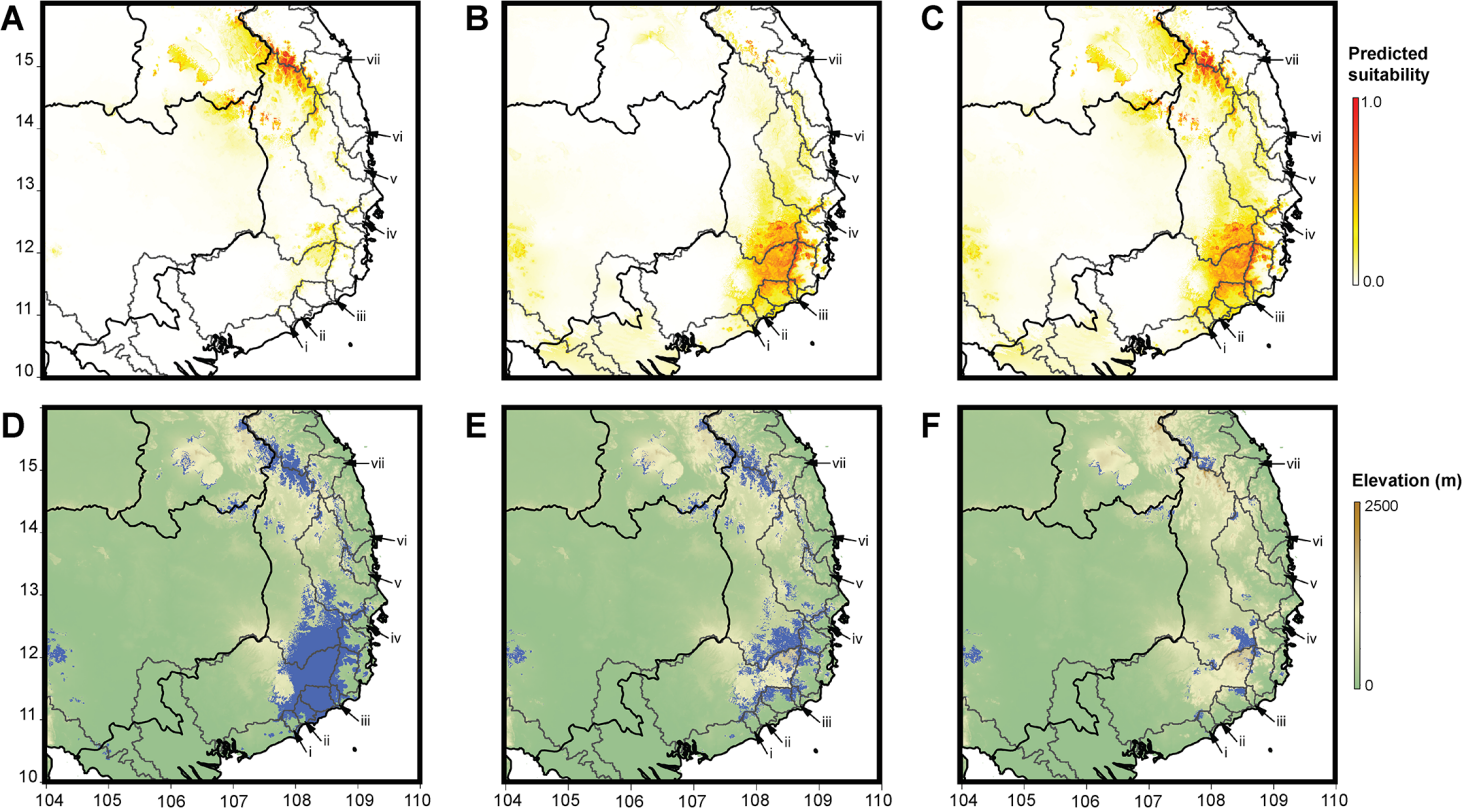
Ecological niche suitability for frogs in the *Leptolalax applebyi* group (A) MaxEnt ecological niche suitability for frogs in the *Leptolalax applebyi* group of the northern region. Grey lines outline the seven drainage basins in which frogs were observed, together with seven additional drainage basins containing areas climatically suitable for the group, marked i–vii. (B) MaxEnt ecological niche suitability for the southern region. (C) Merged MaxEnt ecological niche suitability, based on the maximum of A and B. (D) Area predicted to be climatically suitable for the *L*. *applebyi* group, based on thresholding at 0.333 for the northern region A and 0.144 for the southern region B, and then merging areas. (E) Portion of the area predicted to be climatically suitable for the *L*. *applebyi* group that was covered by Broadleaf Evergreen Forest in 2008 (55.3%). (F) Portion of area predicted to be climatically suitable for the *L*. *applebyi* group and covered by Broadleaf Evergreen Forest in 2008 and that currently falls within protected areas (29.3%)

A large area of southern and central Vietnam and eastern Cambodia was predicted to be climatically suitable for the *L*. *applebyi* group (Fig 6A). The median AUC scores were 0.990 for the northern region (Fig 6A) and 0.983 for the southern region (Fig 6B). Modelling only a single region resulted in a lower AUC score of 0.957, supporting the decision to model the northern and southern regions separately.

Calculated thresholds were 0.333 for the northern region and 0.144 for the southern region. The lower, less conservative, value for the southern region is the result of reduced uncertainty due to the greater number of records for that area. When thresholds were applied, the total area predicted to be climatically suitable for *L*. *applebyi* group species was 30,011 km^2^, centering on the Kon Tum Plateau, northeastern Cambodia and central Vietnam, and the Langbian Plateau, southern Vietnam (Fig 6D). 55.3% of this climatically suitable area was covered by Broadleaf Evergreen Forest in 2008 (Fig 6E), but only 22.4% of the climatically suitable area (29.3% of the portion covered by Broadleaf Evergreen Forest) currently falls within protected areas (Fig 6F).

In addition to the drainage basins where we detected frogs in the *L*. *applebyi* group, there are seven additional watershed basins (labelled i–vii in Fig 6) which each contained over 100 km^2^ predicted to be climatically suitable for the species group. The climatically suitable area of Broadleaf Evergreen forest in these watershed basins ranges from 163 km^2^ (basin ii in Fig 6) to 418 km^2^ (basin vii in Fig 6). However, only basins i and vi have large areas of this forest falling within protected areas (58% and 26% of the climatically suitable forested area respectively). There is 1% or less suitable Broadleaf Evergreen Forest within protected areas in the other five basins.

**Fig 7:**
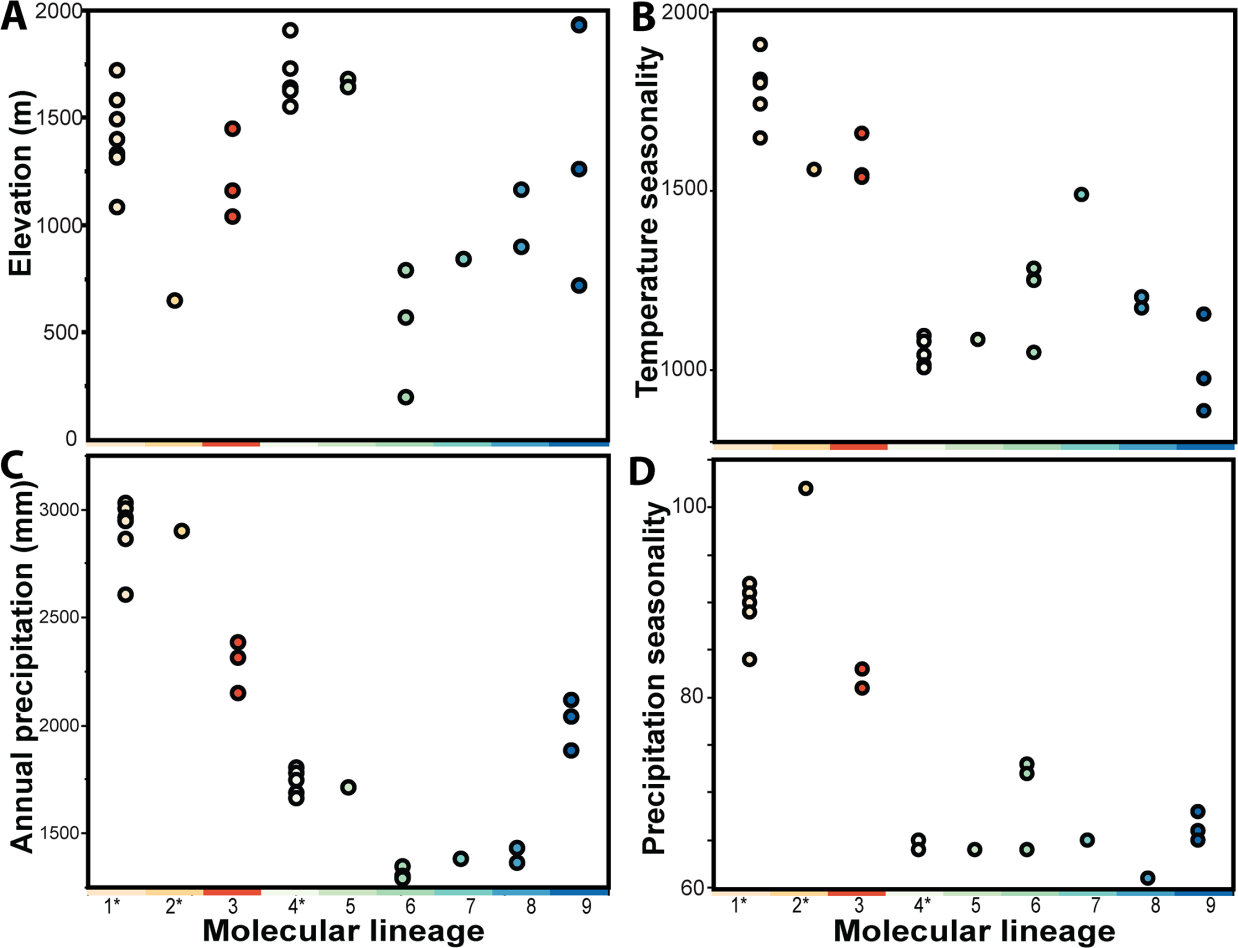
Bioclimatic variables at collection localities for frogs in each of the nine molecular lineages identified in *Leptolalax applebyi* group. (A) elevation above sea level (m), (B) temperature seasonality, (C) annual precipitation (mm), (D) precipitation seasonality. Currently recognized species are marked with an asterix (1 = *L*. *applebyi*, 2 = *L*. *melicus*, 4 = *L*. *bidoupensis*).

The 31 sites at which frogs in the *L*. *applebyi* group were collected varied in terms of elevation above sea level (m), temperature seasonality, annual precipitation (mm), and precipitation seasonality (Fig 7). In particular, the northern lineages (*L*. *applebyi*, *L*. *melicus* and lineage 3) appear to have overall greater temperature seasonality, annual precipitation and precipitation seasonality than the southern lineages (*L*. *bidoupensis* and lineages 5–9; Fig 7).

## Discussion

This study provides evidence of a significant underestimation of diversity in the *Leptolalax applebyi* group of the Central Highlands in Vietnam and northeastern Cambodia. Using a combination of molecular, morphometric, and acoustic data, we conclude that at least two-thirds of the diversity of the group remains hidden within morphologically cryptic lineages. In addition to the three known species, our study identified six well-differentiated lineages on the basis of concordant mtDNA and nuDNA data. Overall, we obtained well-resolved genetic diversification among all lineages identified, with generally concordant mtDNA and nuDNA data. The only evidence of incomplete lineage sorting was in one of the more slowly evolving nuclear genes (NCX), which is typical of recently diversified species [73–75]. The percent uncorrected pairwise divergence at the 16S rRNA mitochondrial gene fragment examined ranged from 4.48–11.21%, and although variable, uncorrected pairwise divergences of 3–5% at the 16S rRNA gene are usually representative of differentiation at the species level in amphibians [76]. Those species most similar in external morphology, such as lineages 6 and 8, and *L*. *bidoupensis* and lineage 9, were not resolved sister species in the molecular phylogenies, further supporting their validity as separate evolutionary lineages and candidate species worthy of further investigation. We refrain from formally delineating and describing these lineages as distinct species here, as the clarification of the taxonomic status of the lineages requires thorough integrative revision.

It is likely that diversification within the species group and apparent microendemism has been driven by the low vagility of the species group and specific habitat requirements. Mountain ridges and valleys appear to present barriers to dispersal for the frogs, with several lineages apparently restricted to specific basins, and another restricted to the northern and southern portions of a larger basin, separated by a valley. Amphibians are poor dispersers in general [77], and adults of frogs in the *L*. *applebyi* group are an order of magnitude smaller than most other *Leptolalax* species. Small-bodied frogs tend to move smaller distances that large-bodied frogs [78], and in general, small body size in animals, including frogs, is correlated with range size [79, 80]. Smaller amphibians also lose water at a higher rate than larger amphibians (e.g. [81]). This physiological constraint may make them less likely to successfully traverse over the more exposed mountain ridges. Dispersal events during the larval stage are also likely to be limited. Although tadpoles are not yet known, frogs in this group have been observed calling from seeps and soaks at the headwaters of small streams, and not the larger streams that most *Leptolalax* in the region breed in.

The relative morphological stasis in the group is likely to have been driven by the use of non-morphological species recognition and mate selection. We found evidence of considerable variation in the structure of advertisement calls within the group, with the number of notes, the presence, duration and number of pulses in an introductory note, and pulse number in non-introductory notes differing among lineages. Such differences in advertisement calls are often taken as evidence of prezygotic reproductive barriers [51, 82–84]. Although our sample sizes were small and comparisons were complicated by the effect of body size and temperature, our acoustic data corroborates molecular patterns. Existing morphotypes may have also been favored by the relatively stable environmental conditions such as those encountered in the leaf-litter in evergreen forests [9, 85].

We predict that the *Leptolalax applebyi* group is likely to have historically occurred over a large area (~30,000 km^2^), centering on the Central Highlands, particularly the Kon Tum Plateau, northeastern Cambodia and central Vietnam, and the Langbian Plateau, southern Vietnam. Originally almost completely forested [86], evergreen forest is likely to have covered a much larger area historically, and it is apparent that a considerable portion of the range of the group has already been lost due to deforestation. Active deforestation is still occurring in the Central Highlands [87], with forest being converted into agricultural land to grow cash crop plantations (such as rubber, coffee and tea) [88,89]. While secondary forests and plantation forests may constitute viable habitats for widespread, generalist amphibian species, highly disturbed habitats tend to be dominated by only a few species of amphibian [27,90], and are unlikely to be suitable for evergreen forest specialists such as the *L*. *applebyi* group. Assuming all unprotected Broadleaf Evergreen Forest is lost or significantly degraded in the future, less than a quarter of the predicted historical range of the species group is likely to remain in the future, all within current protected areas.

Our modelling allowed us to predict not only the distribution of the species group, but also potential diversity in the group not sampled during our study. The predicted distribution of the *Leptolalax applebyi* group extended considerably into seven watershed basins and that we did not sample, and given the association of identified lineages with watershed basins, we speculate that there may be a number of additional lineages that we did not sample. While the predicted distribution of the species group in these watershed basins is still substantially forested (>50% Broadleaf Evergreen Forest) in two of the seven watershed basins, in five of the seven watershed basins less than 1% this forest is currently protected.

Given the rate of deforestation occurring in the region, it is quite possible that, if *L*. *applebyi* group frogs do exist in these areas, they may not do so for long, or they may have already been extirpated. Recent estimates of the proportion of undiscovered extinct species ranged from 0.15 to 0.59, depending on the taxonomic group and geographic area considered [91]. In Central America, there are already documented cases of amphibian species being driven to extinction before they are described [92–94]. Although no Southeast Asian amphibian is known to have been driven to extinction [95], it is possible that extinctions of undescribed amphibian species have gone unnoticed. In order to prevent this from happening (perhaps further) in the Central Highlands of Vietnam, future survey efforts for the *L*. *applebyi* group should focus on these more poorly surveyed and highly threatened watershed basins to the east of the Central Highlands, including parts of Quang Ngai, Binh Dinh, Phu Yen, and Binh Thuan Provinces.

There is an urgent need to gain a greater understanding of the biodiversity of the Central Highlands of Vietnam and beyond in order to prioritize biodiversity conservation, particularly that under threat from ongoing deforestation. Prioritizing habitats for conservation often relies on estimation of species richness and endemism, and therefore an understanding of species boundaries and distributions. It is apparent from this, and other studies from around the world that current estimates of amphibian diversity based on morphology alone are misleading. When amphibian species groups have been examined in light of morphological and molecular and/or acoustic data, estimated species numbers have increased dramatically (eg. from 60 to 129 species, [96]; 16 to 47 [8]). Because naming species is necessary for species-based conservation [14], accurate alpha taxonomy is essential to effective conservation management.

## Supporting Information

S1 Table List of voucher specimens and GenBank accession numbers for all DNA sequences included in the analysis. (DOCX)

S2 Table List of voucher specimens examined morphologically. (DOCX)

S3 Table List of advertisement calls analysed and associated voucher specimens. (DOCX)

S4 Table Rotated factor loadings of a Principal Component Analysis of morphometric comparisons among males of the *L*. *applebyi* group. (DOCX)

S5 Table Factor loadings of a Principal Component Analysis of bioclimatic variables in the region. (DOCX)
