# Supplementary material for: Undiagnosed Cryptic Diversity in Small, Microendemic Frogs (Leptolalax) from the Central Highlands of Vietnam
Source: PLoS One. 2015 May 28;10(5):e0128382. doi: 10.1371/journal.pone.0128382 (PMC4447284; doi:10.1371/journal.pone.0128382)
Supplement: S1 Table — (DOCX) [file pone.0128382.s001.docx]

**Table S1.** List of voucher specimens and GenBank accession numbers for all DNA sequences included in the analysis.

| **Species** | **Locality** | **Voucher #** | **16S** | **Cytb** | **NTF3** | **SLC8A3** | **NCX** |
| --- | --- | --- | --- | --- | --- | --- | --- |
| *Leptolalax applebyi* | Quang Nam Province, Vietnam | AMS R 171703 | HM133597 | KR018001 | KR018026 | KR018081 | KR018053 |
| *Leptolalax applebyi* | Kon Tum Province, Vietnam | AMS R 173778 | KR018108 | KR018002 | KR018027 | KR018082 | KR018054 |
| *Leptolalax bidoupensis* | Lam Dong Province, Vietnam | AMS R 173133 | HQ902880 | KR018003 | KR018028 | KR018083 | KR018055 |
| *Leptolalax bidoupensis* | Lam Dong Province, Vietnam | NCSM 77321 | HQ902883 | KR018004 | KR018029 | KR018084 | KR018056 |
| *Leptolalax bidoupensis* | Lam Dong Province, Vietnam | AMS R 173134 | HQ90288 | KR018005 | KR018030 | KR018085 | KR018057 |
| *Leptolalax bidoupensis* | Lam Dong Province, Vietnam | NCSM 77320 | HQ902882 | KR018006 | KR018031 | KR018086 | KR018058 |
| *Leptolalax melicus* | Ratanakiri Province, Cambodia | MVZ 258197 | HM133599 | - | KR018032 | KR018087 | KR018059 |
| *Leptolalax melicus* | Ratanakiri Province, Cambodia | MVZ 258198 | HM133600 | - | KR018033 | KR018088 | KR018060 |
| *Leptolalax melicus* | Ratanakiri Province, Cambodia | MVZ 258199 | HM133601 | - | KR018034 | - | KR018061 |
| *Leptolalax* sp. (Lineage 3) | Gia Lai Province, Vietnam | AMS R 176454 | KR018109 | KR018007 | KR018035 | KR018089 | KR018062 |
| *Leptolalax* sp. (Lineage 3) | Gia Lai Province, Vietnam | AMS R 176463 | KR018110 | KR018008 | KR018036 | KR018090 | KR018063 |
| *Leptolalax* sp. (Lineage 3) | Gia Lai Province, Vietnam | AMS R 176467 | KR018111 | KR018009 | KR018037 | KR018091 | KR018064 |
| *Leptolalax* sp. (Lineage 5) | Lam Dong Province, Vietnam | UNS00510 | KR018112 | KR018010 | KR018038 | KR018092 | KR018065 |
| *Leptolalax* sp. (Lineage 5) | Lam Dong Province, Vietnam | UNS00512 | KR018113 | KR018011 | KR018039 | KR018093 | KR018066 |
| *Leptolalax* sp. (Lineage 6) | Binh Thuan Province, Vietnam | IEBR A.2014.15 | KR018114 | KR018012 | KR018040 | KR018094 | KR018067 |
| *Leptolalax* sp. (Lineage 6) | Binh Thuan Province, Vietnam | AMNH A191762 | KR018115 | KR018013 | KR018041 | KR018095 | KR018068 |
| *Leptolalax* sp. (Lineage 6) | Binh Thuan Province, Vietnam | IEBR A.2014.16 | KR018116 | KR018014 | KR018042 | KR018096 | KR018069 |
| *Leptolalax* sp. (Lineage 6) | Binh Thuan Province, Vietnam | AMNH A191765 | KR018117 | KR018015 | - | KR018097 | KR018070 |
| *Leptolalax* sp. (Lineage 7) | Dak Lak Province, Vietnam | AMS R 177663 | KR018118 | KR018016 | KR018043 | KR018098 | KR018071 |
| *Leptolalax* sp. (Lineage 8) | Ninh Thuận Province, Vietnam | AMS R 177660 | KR018119 | KR018017 | KR018044 | KR018099 | KR018072 |
| *Leptolalax* sp. (Lineage 8) | Ninh Thuận Province, Vietnam | ZFMK 96600 | KR018120 | KR018018 | KR018045 | KR018100 | KR018073 |
| *Leptolalax* sp. (Lineage 9) | Dak Nong Province, Vietnam | UNS00515 | KR018121 | KR018019 | KR018046 | KR018101 | KR018074 |
| *Leptolalax* sp. (Lineage 9) | Dak Nong Province, Vietnam | UNS00517 | KR018122 | KR018020 | KR018047 | KR018102 | KR018075 |
| *Leptolalax firthi* | Quang Nam Province, Vietnam | AMS R 171714 | JQ739203 | KR018021 | KR018048 | KR018103 | KR018076 |
| *Leptolalax aereus* | Savannakhet Province, Laos | SAM R64242 | KR018123 | KR018022 | KR018049 | KR018104 | KR018077 |
| *Leptolalax bourreti* | Lao Cai Province, Vietnam | AMS R 177673 | KR018124 | KR018023 | KR018050 | KR018105 | KR018078 |
| *Oreolalax sterlingae* | Lao Cai Province, Vietnam | AMS R 177528 | KR018125 | KR018024 | KR018051 | KR018106 | KR018079 |
| *Leprobrachium* cf. *chapaense* | Lao Cai Province, Vietanm | AMS R 171623 | KR018126 | KR018025 | KR018052 | KR018107 | KR018080 |
