# Supplementary material for: Undiagnosed Cryptic Diversity in Small, Microendemic Frogs (Leptolalax) from the Central Highlands of Vietnam
Source: PLoS One. 2015 May 28;10(5):e0128382. doi: 10.1371/journal.pone.0128382 (PMC4447284; doi:10.1371/journal.pone.0128382)
Supplement: S2 Table — (DOCX) [file pone.0128382.s002.docx]

**Table S2.** List of voucher specimens examined morphologically.

*Leptolalax applebyi:* Vietnam, Quang Nam Province, Song Thanh Proposed Nature Reserve (AMS R 171703–171707; type specimens). Vietnam, Kon Tum Province, Ngoc Linh Nature Reserve (AMS R 173635, AMS R 177637, UNS 00462/AMS R 173776, AMS R 173777, AMS R 173778, UNS 00465/AMS R 173780, AMS R 176528, UNS 00464/AMS R 176529, UNS 00465/AMS R 176530, AMS R 176531–176533).

*Leptolalax bidoupensis*: Vietnam, Lam Dong Province, Bidoup–Nui Ba National Park (AMS R 173133, UNS 00101/AMS R 173135, UNS 00102/AMS R 173137, AMS R 173134, AMS R 173136, NCSM 77320, NCSM 77321, NCSM 77322; type specimens); (AMS R 173511, AMS R 173512, UNS 00522–00525, ZFMK 96595–96597).

*Leptolalax melicus*: Cambodia, Ratanakiri Province, Virachey National Park (MVZ 258074–258077, MVZ 258197–258199; type specimens).

*Leptolalax sp*.: Vietnam, Gia Lai Province (AMS R 176452–176467). Vietnam, Lam Dong Province (AMS R 177657–177659, UNS00510–00512, ZFMK 96598–96599). Vietnam, Binh Thuan Province (AMNH A191761–191770, IEBR A.2014.14–2014.22). Vietnam, Dak Lak Province (AMS R 177663–177665, UNS00518–00521). Vietnam, Ninh Thuan Province (AMS R 177660, UNS00513, UNS00514, ZFMK 96600). Vietnam, Dak Nong Province (AMS R 177661, AMS R 177662, AMS R 177666, UNS 00526, UNS 00527, UNS00515–00517, ZFMK 96601–96603).
