# Supplementary material for: Undiagnosed Cryptic Diversity in Small, Microendemic Frogs (Leptolalax) from the Central Highlands of Vietnam
Source: PLoS One. 2015 May 28;10(5):e0128382. doi: 10.1371/journal.pone.0128382 (PMC4447284; doi:10.1371/journal.pone.0128382)
Supplement: S3 Table — (DOCX) [file pone.0128382.s003.docx]

**Table S3.** List of advertisement calls analysed and associated voucher specimens.

| Species | Specimen |
| --- | --- |
| *Leptolalax applebyi* | AMS R 173635 |
| *Leptolalax applebyi* | AMS R 171703 |
| *Leptolalax applebyi* | No voucher |
| *Leptolalax bidoupensis* | AMS R 173133 |
| *Leptolalax bidoupensis* | AMS R 173135/UNS 00101 |
| *Leptolalax bidoupensis* | No voucher |
| *Leptolalax melicus* | MVZ 258198 |
| *Leptolalax melicus* | MVZ 258199 |
| *Leptolalax* sp. (Lineage 3) | No voucher |
| *Leptolalax* sp. (Lineage 3) | AMS R 176459 |
| *Leptolalax* sp. (Lineage 3) | AMS R 176466 |
| *Leptolalax* sp. (Lineage 3) | No voucher |
| *Leptolalax* sp. (Lineage 3) | No voucher |
| *Leptolalax* sp. (Lineage 3) | AMS R 176467 |
| *Leptolalax* sp. (Lineage 3) | No voucher |
| *Leptolalax* sp. (Lineage 5) | ZFMK 96598 |
| *Leptolalax* sp. (Lineage 5) | ZFMK 96599 |
| *Leptolalax* sp. (Lineage 5) | UNS00510 |
| *Leptolalax* sp. (Lineage 6) | AMNH A191770 |
| *Leptolalax* sp. (Lineage 8) | UNS00513 |
| *Leptolalax* sp. (Lineage 8) | AMS R 177660 |
| *Leptolalax* sp. (Lineage 9) | UNS 00526 |
| *Leptolalax* sp. (Lineage 9) | UNS00516 |
| *Leptolalax* sp. (Lineage 9) | AMS R 177666 |
| *Leptolalax* sp. (Lineage 9) | UNS 00527 |
