# Supplementary material for: Undiagnosed Cryptic Diversity in Small, Microendemic Frogs (Leptolalax) from the Central Highlands of Vietnam
Source: PLoS One. 2015 May 28;10(5):e0128382. doi: 10.1371/journal.pone.0128382 (PMC4447284; doi:10.1371/journal.pone.0128382)
Supplement: S4 Table — (DOCX) [file pone.0128382.s004.docx]

**Table S4. Rotated factor loadings of a Principal Component Analysis of morphometric comparisons among males of the *L. applebyi* group.**

|  | Factor 1 | Factor 2 |
| --- | --- | --- |
| SVL | **0.8838123** | 0.3809014 |
| HDL | 0.3723791 | 0.5640768 |
| HDW | **0.8643034** | 0.3947372 |
| SNT | **0.8350739** | 0.2580975 |
| EYE | 0.7571414 | 0.4301991 |
| IOD | **0.819156** | 0.2294101 |
| TMP | **0.8285685** | 0.150239 |
| TEY | 0.5582954 | 0.5376509 |
| TIB | **0.8321825** | 0.3971019 |
| PEC | 0.1463143 | **0.8223919** |
| FEM | 0.3065374 | 0.5656512 |
| HUM | 0.2096474 | 0.7870264 |
