# Supplementary material for: Undiagnosed Cryptic Diversity in Small, Microendemic Frogs (Leptolalax) from the Central Highlands of Vietnam
Source: PLoS One. 2015 May 28;10(5):e0128382. doi: 10.1371/journal.pone.0128382 (PMC4447284; doi:10.1371/journal.pone.0128382)
Supplement: S5 Table — (DOCX) [file pone.0128382.s005.docx]

**Table S5. Factor loadings of a Principal Component Analysis of bioclimatic variables in the region.**

| **Bioclimatic Variable** | **PC1** | **PC2** | **PC3** | **PC4** | **PC5** | **PC6** | **PC7** | **PC8** |
| --- | --- | --- | --- | --- | --- | --- | --- | --- |
| Annual Mean Temperature | -0.02 | 0.00 | -0.08 | 0.01 | 0.06 | 0.35 | 0.04 | -0.03 |
| Mean Diurnal Range | 0.01 | 0.00 | 0.00 | 0.03 | -0.11 | -0.02 | -0.02 | 0.31 |
| Isothermality | 0.00 | -0.01 | 0.01 | -0.01 | 0.00 | -0.02 | 0.00 | -0.02 |
| Temperature Seasonality | 0.33 | 0.93 | 0.05 | -0.10 | -0.08 | 0.05 | 0.03 | -0.03 |
| Max Temp, Warmest Month | -0.01 | 0.01 | -0.09 | 0.02 | -0.02 | 0.37 | 0.00 | 0.25 |
| Min Temp, Coldest Month | -0.03 | -0.01 | -0.07 | -0.06 | 0.17 | 0.34 | 0.03 | -0.30 |
| Temperature Annual Range | 0.02 | 0.02 | -0.02 | 0.07 | -0.19 | 0.03 | -0.02 | 0.55 |
| Mean Temp, Wettest Q | -0.02 | 0.00 | -0.06 | 0.02 | 0.04 | 0.35 | 0.05 | -0.02 |
| Mean Temp, Driest Q | -0.02 | -0.01 | -0.08 | -0.03 | 0.09 | 0.36 | 0.05 | -0.06 |
| Mean Temp, Warmest Q | -0.02 | 0.01 | -0.08 | 0.00 | 0.06 | 0.36 | 0.04 | -0.01 |
| Mean Temp, Coldest Q | -0.02 | -0.01 | -0.08 | 0.00 | 0.07 | 0.36 | 0.04 | -0.03 |
| Annual Precipitation | 0.72 | -0.35 | 0.02 | -0.42 | -0.38 | 0.10 | 0.15 | -0.03 |
| Precipitation, Wettest Month | 0.21 | -0.02 | -0.11 | 0.21 | 0.44 | -0.16 | 0.81 | 0.15 |
| Precipitation, Driest Month | 0.00 | 0.00 | 0.02 | -0.06 | 0.05 | -0.04 | 0.02 | -0.16 |
| Precipitation Seasonality | 0.01 | 0.01 | -0.04 | 0.07 | 0.01 | 0.04 | -0.05 | 0.04 |
| Precipitation, Wettest Q | 0.53 | -0.07 | -0.35 | 0.45 | 0.35 | -0.07 | -0.49 | -0.05 |
| Precipitation, Driest Q | 0.01 | 0.01 | 0.07 | -0.24 | 0.14 | -0.16 | 0.03 | -0.50 |
| Precipitation, Warmest Q | 0.19 | -0.08 | 0.90 | 0.25 | 0.21 | 0.18 | -0.08 | 0.04 |
| Precipitation, Coldest Q | 0.00 | 0.00 | 0.02 | -0.66 | 0.61 | -0.05 | -0.23 | 0.36 |

To account for correlations among the data, we performed a principal components analysis on the 19 WorldClim “bioclimatic variables”[55]. We defined our study area as a region 6° on each side containing our survey sites, from 104–110°E and 10–16°N. The first eight principal components together accounted for 99.98% of the variance in the data
